# Supplementary material for: Discovery of the Environmental Factors Affecting Urban Dwellers’ Mental Health: A Data-Driven Approach
Source: Int J Environ Res Public Health. 2020 Nov 5;17(21):8167. doi: 10.3390/ijerph17218167 (PMC7672565; doi:10.3390/ijerph17218167)
Supplement: Supplementary file 1 [file ijerph-17-08167-s001.zip › ijerph-918674-supplementary.docx]

**Supplementary**

**Table S1.** Greater London Data Sets 2015.

| **Happiness** |
| --- |
| Employment: Primary and& Utilities |
| Employment: Manufacturing |
| Employment: Construction |
| Employment: Wholesale |
| Employment: Retail |
| Employment: Transportation and Storage |
| Employment: Accommodation and Food Service Activities |
| Employment: Information and Communication |
| Employment: Financial and Insurance Activities |
| Employment: Professional, Real Estate, Scientific and Technical Activities |
| Employment: Administrative and Support Service Activities |
| Employment: Public Admin and Defense |
| Employment: Education |
| Employment: Health |
| Employment: Arts, Entertainment and Recreation |
| Employment: Other Services |
| Economics: Accommodation and Food Service Activities |
| Economics: Administrative and Support Service Activities |
| Economics: All Sectors |
| Economics: Arts, Entertainment and Recreation |
| Economics: Construction |
| Economics: Education |
| Economics: Financial and Insurance Activities |
| Economics: Health |
| Economics: Information and Communication |
| Economics: Manufacturing |
| Economics: Other Services |
| Economics: Primary and Utilities |
| Economics: Professional, Real Estate, Scientific and Technical Activities |
| Economics: Public Admin and Defense |
| Economics: Retail |
| Economics: Transportation and Storage |
| Economics: Wholesale |
| Home Fire Safety Visits (LFEPA) |
| Home Fire Safety Visits (Partnership) |
| Housing Benefit Caseload |
| Housing Benefit Rates (per 100 aged 18+) |
| New Migrant GP Registrations |
| Short: Term Migration |
| Population |
| Births |
| International |
| Domestic |
| Population by Nationality (British) |
| Population by Nationality (Non: British) |
| Population by Nationality (European Union) |
| Population by Nationality (Asia) |
| Inland Area (Hectares) |
| Proportion of Population Aged 0–15, 2015 |
| Proportion of Population of Working Age, 2015 |
| Proportion of Population Aged 65 And Over, 2015 |
| Net Internal Migration (2015) |
| Net International Migration (2015) |
| Net Natural Change (2015) |
| % of Resident Population Born Abroad (2015) |
| All Fires |
| Chimney Fires |
| Dwelling Fire Injuries |
| Dwelling Fire Fatalities |
| Dwelling Fires |
| Fire Related Fatalities |
| Fire Related Injures |
| Fires in Non: Residential Buildings |
| Fires in Other Residential Buildings |
| Secondary Fires |
| Primary Fires |

**Table S2.** Greater London Data Sets 2016.

| **Happiness** |
| --- |
| Home Fire Safety Visits (LFEPA) |
| Home Fire Safety Visits (Partnership) |
| New Migrant GP Registrations |
| Short-Term Migration |
| Population |
| Births |
| International |
| Domestic |
| Population by Nationality(British) |
| Population by Nationality(Non-British) |
| Population by Nationality(European Union) |
| Population by Nationality(Asia) |
| Inland Area (Hectares) |
| All Fires |
| Chimney Fires |
| Dwelling Fire Injuries |
| Dwelling Fire Fatalities |
| Dwelling Fires |
| Fire Related Fatalities |
| Fire Related Injures |
| Fires in Non-Residential Buildings |
| Fires in Other Residential Buildings |
| Secondary Fires |
| Primary Fires |
| EU Referendum Results: Electorate |
| EU Referendum Results: Expected Ballots |
| EU Referendum Results: Verified Ballot Papers |
| EU Referendum Results: Turnout (%) |
| EU Referendum Results: Votes Cast |
| EU Referendum Results: Valid Votes |
| EU Referendum Results: Remain |
| EU Referendum Results: Leave |
| EU Referendum Results: Rejected Ballots |
| EU Referendum Results: No Official Mark |
| EU Referendum Results: Voting for Both Answers |
| EU Referendum Results: Writing or Mark |
| EU Referendum Results: Unmarked or Void |
| EU Referendum Results: Remain (%) |
| EU Referendum Results: Leave (%) |
| EU Referendum Results: Rejected (%) |

**Table S3.** Greater London Data Sets 2017.

| **Happiness** |
| --- |
| Home Fire Safety Visits (Lfepa) |
| Home Fire Safety Visits (Partnership) |
| New Migrant GP Registrations |
| Short-Term Migration |
| Population by Nationality (British) |
| Population by Nationality (Non-British) |
| Population by Nationality (European Union) |
| Population by Nationality (Asia) |
| Inland Area (Hectares) |
| Proportion Of Households Fuel Poor (%) |
| Average Domestic Electricity Consumption per Household (Kwh) |
| Average Domestic Gas Consumption |
| Household Waste Recycling Rates |
| PLG Cars: Company |
| PLG Cars: Private |
| Plg: Other Company |
| Plg: Other Private |
| Total PLG |
| Exempt Disabled: Cars |
| Exempt Disabled: Others |
| Gla Population Estimate |
| Gla Household Estimate |
| Population Density (Per Hectare) |
| Average Age |
| All Fires |
| Dwelling Fire Injuries |
| Dwelling Fires |
| Fire Related Injures |
| Fires in Non-Residential Buildings |
| Fires in Other Residential Buildings |
| Secondary Fires |
| Primary Fires |
| Mean Average Value of Help To Buy Loans (2017) |
